# Reduction of maximal false lumen area ratio by interactive cannulation perfusion in DeBakey type I acute aortic dissection repair

**DOI:** 10.34172/jcvtr.025.33215

**Published:** 2025-06-28

**Authors:** Qin Jiang, Shanshan Lin, Xiaoxiao Gou, Tao Yu, Keli Huang, Shengshou Hu

**Affiliations:** ^1^Department of Cardiac Surgery, Sichuan Provincial People’s Hospital, Affiliated Hospital of University of Electronic Science and Technology, Chengdu, China; ^2^Department of Cardiac Surgery, Fuwai Hospital, Chinese Academy of Medical Sciences and Peking Union Medical College, Beijing, China

**Keywords:** Interactive cannulation perfusion mode, Maximal false lumen area, Acute aortic dissection, Inflammation response, Anaerobic metabolism

## Abstract

**Introduction:**

Acknowledging lacking of recognition on postoperative aortic remodeling by intraoperative transition of cannulation perfusion mode during the open repair surgery of DeBakey type I acute aortic dissection (AAD), this study aims to investigate the effect of interactive cannulation strategy on the maximum false lumen area (MFLA) ratio.

**Methods:**

A total of 321 AAD patients were retrospectively reviewed from March 2017 to March 2023, of which 166 patients receiving peripheral cannulation (PC, right axillary and femoral artery) and 155 patients receiving peripheral-to-centric cannulation (PCC, transition from right axillary and femoral artery to one branch of the tetrafurcated graft). The primary outcome was postoperative MFLA ratio in descending thoracic aorta. Secondary outcomes were postoperative inflammation response and anaerobic metabolism, hepatorenal dysfunction, and the ostium condition of branch artery of abdominal aorta involved by false lumen.

**Results:**

There was a lower postoperative MFLA ratio in PCC group than that in PC group, respectively (0.36±0.11 vs. 0.44±0.13, *P*<0.001). The abdominal branch arteries involved by false lumen was also deceased in PCC group. There was also a lower serum inflammation response (24 hours, hr-CRP: 111.8±14.1mg/L vs. 116.8±15.0mg/L, *P*=0.002; IL-6: 104.4±49.9pg/ml vs. 124.0±50.1pg/ml, *P*<0.001), anaerobic metabolism (8 hours, lactate: 8.3±1.5mmol/L vs. 8.8±1.6mmol/L, *P*=0.002), impaired liver function (15.5% vs. 39.8%, *P*<0.001) and need for renal replacement therapy (10.3% vs. 20.5%, *P*=0.012) in PCC group than those in PC group.

**Conclusion:**

Interactive cannulation with prompt transition from peripheral artery to centric perfusion during surgical repair of AAD was associated with the reduction of MFLA and hepatorenal dysfunction.

## Introduction

 DeBakey type I acute aortic dissection (AAD) has been widely recognized as the indication of emergency procedure in cardiovascular surgical community. Comprehensive strategies for AAD repair improve the survival rate in the early period, but organ malperfusion resulting from the impaired ostium of branch arteries complicating AAD poses a unnegligible challenge and management dilemma.^1-2^ There is still a high incidence of postoperative organ mal-perfusion when residual false lumen compresses against the ostium of true lumen in the descending thoracic aorta (DTA) even through perfect proximal aorta reconstruction, which portends high risk of hepatic dysfunction and renal replacement therapy.^3-4^

 Systematic artery cannulation plays a vital role in the repair of AAD with malperfusion. At present, the more commonly used is peripheral artery cannulation including axillary and femoral artery. Axillary artery cannulation is relatively safe, but associated with the risk of limited flow rate, intraoperative vascular injury and occlusion of vertebral artery orifice. Femoral artery cannulation via retrograde perfusion is easily established but results in malperfusion of vital organs and embolic stroke. Additionally, direct centric cannulation via ascending aorta or aortic arch provides alternative perfusion mode but not widespread applied in the clinical practice due to the risk of exacerbating potential vascular rupture regardless of the rapid establishment of cardiopulmonary bypass (CPB).^5^

 Granted that there are possible shortcomings in peripheral cannulation and potential advantages in centric cannulation, we postulated that the interactive cannulation strategy, which initiated from peripheral artery and then converted to centric branch cannulation (PCC) via one branch of four-branched graft after completing distal anastomosis, rendered a lower postoperative maximum false lumen area (MFLA) ratio in DTA by computed tomography angiography (CTA) compared with peripheral cannulation (PC).

## Materials and Methods

###  Patients and study design

**Figure 1:**
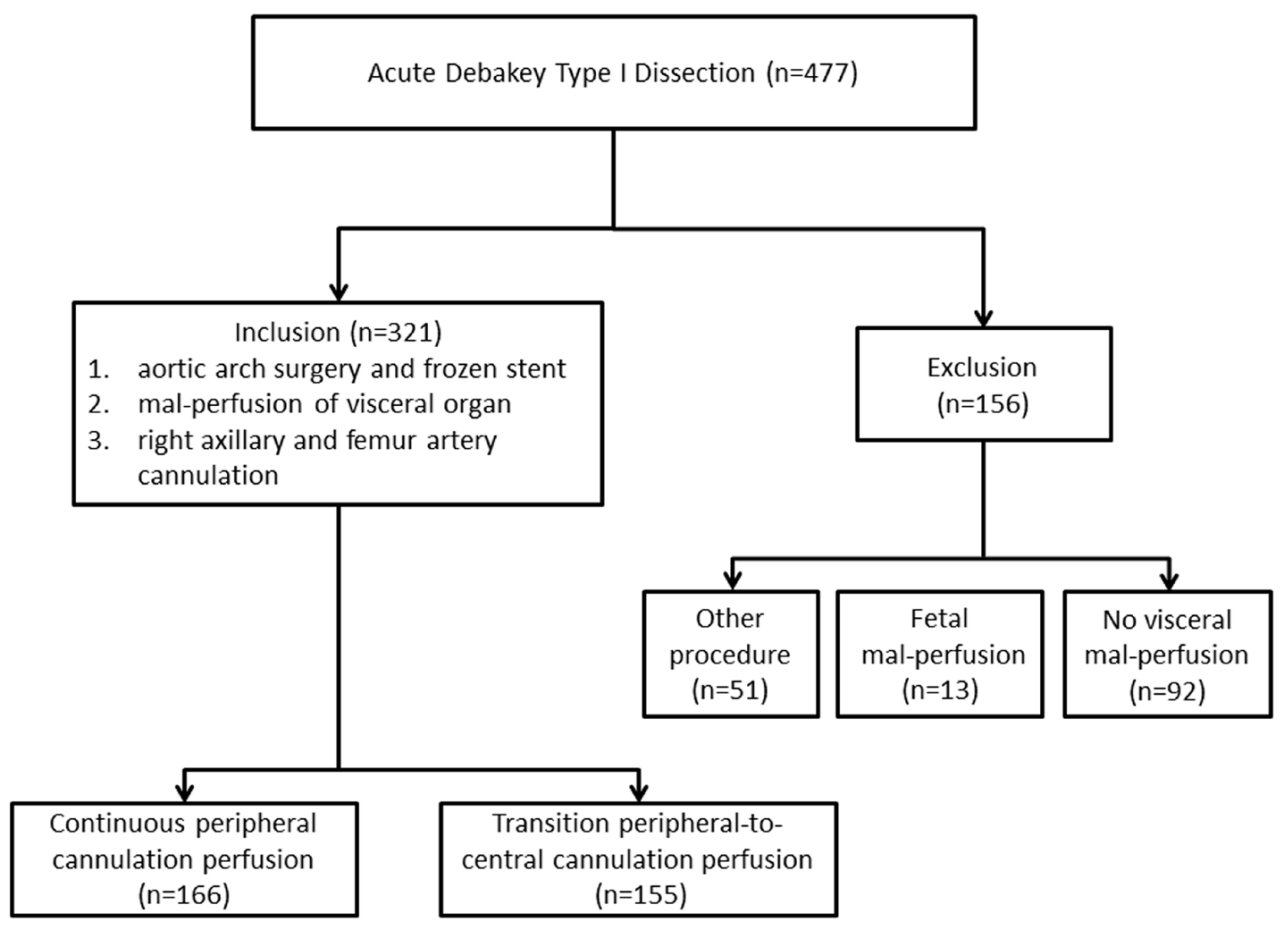
The study flowchart

 Acute DeBakey type I AAD patients who underwent emergency repair procedure were reviewed at the Cardiac Surgical Department of a tertiary hospital from March 2017 to March 2023. The inclusion criteria were any one or more branch artery involved by false lumen such as superior mesenteric artery, coeliac trunk artery and renal artery. The exclusion criteria were other procedure type, other perfusion mode, and fatal liver dysfunction. The included patients were categorized into PCC group and PC group according to the use of perfusion cannulation from peripheral artery to centric cannulation or not. The design of this study is shown in Figure 1. This study was approved by Institutional Medical Ethics Committee (Approval No. 2021215). All the methods were performed on ethical principles outlined in the Declaration of Helsinki and the relevant guidelines and regulations. Due to retrospective nature, the Medicine Ethic Committee Board waived the need to obtain informed consent to participate. The primary outcome was postoperative MFLA ratio in the descending thoracic aorta. The secondary outcomes were in-hospital and 30-day postoperative mortality, the serum inflammation response and metabolism indexes^6^ including high sensitivity C reaction protein(hs-CRP), Interleukin-6(IL-6), lactate, the incidence of hepatorenal dysfunction and the ostium condition of branch artery of abdominal aorta involved by false lumen.

###  Surgical approach and cannulation strategies

**Figure 2:**
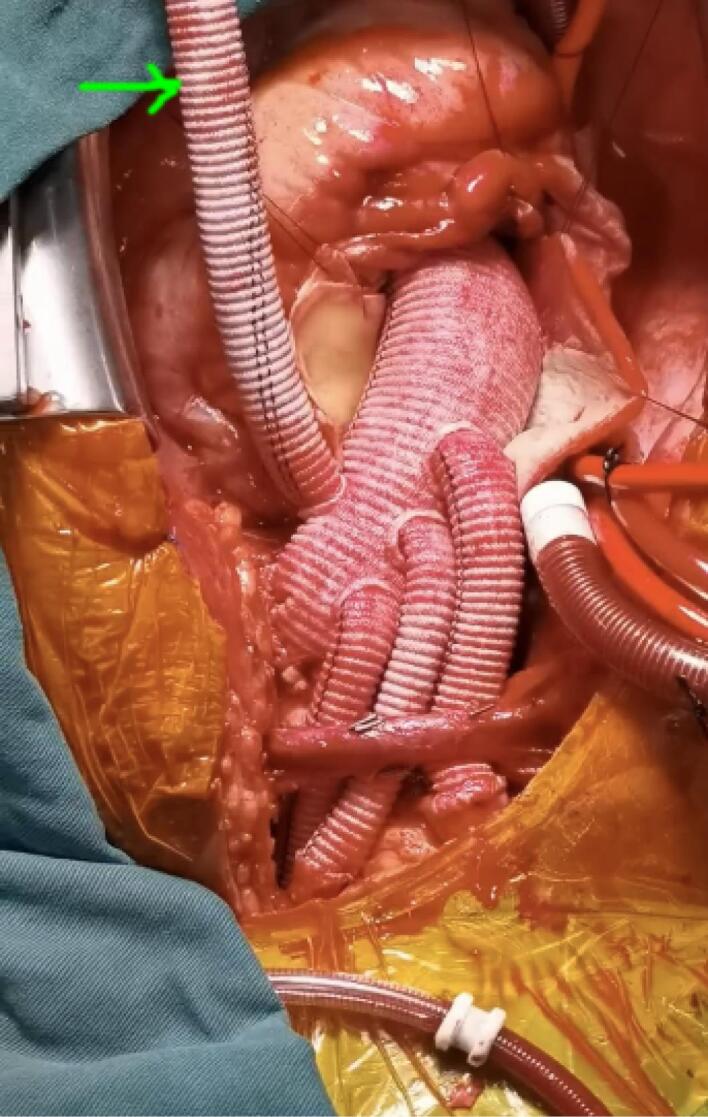
The peripheral-to-centric cannulation perfusion mode in surgical repair of AAD. The centric perfusion was initiated via the perfusion branch of a tetrafurcated graft once distal anastomosis was established. The arrow indicated the centric perfusion via the branch of four-branched graft

 The repair procedure was performed by the same surgical staff under intravenous and inhaled anesthesia. The cannulation method for peripheral vessels was “Seldinger” technique on the normal region as used in thoracoscopic cardiac surgical procedure.^7^ The aortic arch was replaced by using a 4-branched vascular graft (Maquet, Rastatt, Baden-Württemberg, Germany) during hypothermia circulatory arrest (HCA). All the patients were repaired with an intraoperative frozen elephant trunk (CRONUS, MicroPort Scientifc Corporation, Shanghai, China), which resorted to an endovascular stent graft to place around the inward proximal DTA, resulting in true lumen expansion and sealing of entry tears into the false lumen.^8^ In PC group, the perfusion cannulation was maintained on right axillary and femoral artery until the CPB was terminated. In the PCC group, the perfusion cannulation was sequentially transferred from initial femoral artery to the perfusion branch of the tetrafurcated graft once the distal anastomosis was established (Figure 2). Intraoperative cerebral protection was achieved by hypothermia circulatory arrest and selective antegrade perfusion via right axillary artery.

###  Laboratory biomarkers 

 The laboratory biomarkers for systemic inflammatory response, liver and kidney function, were conventionally measured after surgical procedure. The lactate level in the artery was periodically examined through blood gas analysis postoperatively. The hepatic dysfunction was defined as new-onset elevation of any type of serum transaminase or/and bilirubin by > 1.5 times compared with the baseline value. Kidney dysfunction was defined as creatinine > 1.5 mg/dL. Conforming to the implementations of the Kidney Disease Improving Global Outcomes (KDIGO) guidelines, the use of renal replacement therapy (RTT) was considered if suggested by two or more ICU doctors.^9^ The operation data and the hospitalized information were retrieved from electronic medical record.

###  MFLA in DTA

 The ratio of MFLA at the maximum segment in DTA was used to evaluate the postoperative regression of the false lumen, which was calculated from CTA imaging data before admission and at discharge of hospital with freehand annotation using area measurement tool (Neusoft PACS/ RIS Version 5.5 Workstation).^10^ The false lumen area was the result of the aorta area minus to the true lumen. The aortic area (mm^2^) was determined by tracking along the inside contours of the aortic wall using axial CT scans. The branch artery on the abdominal aorta involved by false lumen was also quantified from CTA imaging preoperatively and postoperatively, respectively.

###  Statistical Analysis

 Continuous variables conforming to the normally distributed were demonstrated as mean ± standard deviation (SD) and compared applying the independent t-tests. If continuous variables did not distribute unequally, these were reported as the median and interquartile ranges and were compared with the Mann-Whitney U tests. Categorical variables were reported as counts (percentage) and compared using chi-square tests. In case of small size (n ≤ 5), the Fisher’s exact tests were applicable. Multivariate logistic and forward stepwise regression method identified significant predictors for postoperative MFLA ratio including risk factors such as age, sex, BMI and perfusion mode. All statistical analyses were conducted using SPSS statistic software version 25.0 (SPSS Inc., Chicago, IL, USA) and a two-sided P-value of < 0.05 was defined with a significance level.

## Results

###  Baseline Characteristics 

 A total of 477 patients undergoing DeBakey type I AAD repair surgery were reviewed. Among them, the exclusion causes included the other operation or perfusion type (n = 51), fetal mal-perfusion (n = 13), no visceral mal-perfusion (n = 92). At last, 321 patients were included into this study with 166 patients were cannulated by PC and 155 patients of PCC, respectively. The two groups were similar on the demographic variables and comorbidities including the branch arteries involvement by dissection and MFLA ratio in the descending thoracic aorta (Table 1).

**Table 1:** Baseline characteristics

	**PCC (n=155)**	**PC ** **(n=166)**	***P*** ** value**
Age (years)	51.4 ± 9.5	52.5 ± 9.2	0.322
Male (%)	95 (61.3)	104 (62.7)	0.802
BMI	23.2 ± 2.5	23.1 ± 2.0	0.705
Previous medical history (%)			
Hypertension	129 (83.2)	140 (84.3)	0.880
Dyslipidemia	51 (32.9)	60 (36.1)	0.559
Chronic kidney disease	13 (8.4)	16 (9.6)	0.846
Phaeochromocytoma	1(0.6)	2 (1.2)	1.0
Involved ostium of the branch arteries by intimal flap from CTA imaging (%)			
Coronary artery	17 (11.0)	22 (13.3)	0.609
Brachiocephalic artery	37 (23.9)	36 (21.7)	0.690
Coeliac trunk artery	53 (34.2)	52 (31.3)	0.551
Superior mesenteric artery	47 (30.3)	50 (30.1)	0.969
Renal artery	125 (80.6)	131 (78.9)	0.781
Iliac artery	65 (41.9)	71 (42.8)	0.880
MFLA ratio in descending thoracic aorta	0.70 ± 0.15	0.70 ± 0.13	0.930
Potent false lumen	145 (93.5)	151 (91.0)	0.413
CTA time after onset symptoms (hs)	8.6 ± 2.5	8.6 ± 2.6	0.98
Re-entry tears (%)	122 (78.7)	136 (81.9)	0.468
Total number	2 [1, 3]	2 [1, 3]	0.629
In descending thoracic aorta segment	17 (11.0)	18 (10.8)	0.972
Cardiac function from echocardiography			
LAD (mm)	39.1 ± 6.0	40.0 ± 6.3	0.223
LVEDD (mm)	51.6 ± 6.1	51.3 ± 6.4	0.606
LVEF (%)	56.4 ± 7.1	56.1 ± 7.3	0.758
Pericardial effusion (%)	23 (14.8)	29 (17.5)	0.548
Laboratory tests			
hs-CRP (mg/L)	27.2 ± 12.8	29.9 ± 11.5	0.051
TnI (ng/mL)	0.18 ± 0.29	0.25 ± 0.50	0.162
Lactate (mmol/L)	0.33 ± 0.13	0.34 ± 0.15	0.530
Hb (g/L)	131 ± 19	132 ± 18	0.691
WBC (*10^9^/L)	11.4 ± 2.6	11.4 ± 2.2	0.847
Neutrophil (*10^9^/L)	9.6 ± 2.3	9.6 ± 1.9	0.887
Impaired liver function	23 (14.8)	22 (13.3)	0.683
Creatinine ( > 1.5 mg/dl)	25 (16.1)	31 (18.7)	0.548
Preoperative medications			
Nicardipine	143 (92.3)	150 (90.4)	0.547
Sodium nitroprusside	65 (41.9)	76 (45.8)	0.488
Esmolol	139 (89.7)	146 (88.0)	0.624
Analgesic	132 (85.2)	141 (84.9)	0.956
Laxative	117 (75.5)	123 (74.1)	0.775

Note: PC: peripheral cannulation; PCC: peripheral-to-centric cannulation; BMI: body mass index; CTA: computed tomography angiography; MFLA: maximum false lumen area; LAD: left atrium diameter; LVEDD: left ventricular end-diastolic diameter; LVEF: left ventricular ejection fraction; hs-CRP: high sensitivity C-reactive protein; TnI: troponin I; Hb: hemoglobin; WBC: white blood cell.

###  Intraoperative results

 Ascending aortic replacement concomitant with commissure suspension was dominated at the proximal repair. Modified Carbrol’s procedure was anastomosed with 1cm-length vascular graft between the coronary artery ostium and the aorta graft in the cases who did not directly allow Bentall’s procedure due to excessive tension of anastomosis. The operation type and intraoperative blood transfusion between two groups was not markedly different as listed in Table 2. There was also no remarkable difference in the procedure duration or temperature management between the two groups.

**Table 2:** Operative characteristics

	**PCC (n=155)**	**PC (n=166)**	***P*** ** value**
Surgical type on aortic root (%)			0.987
Remodeling (commissure suspension)	79 (51.0)	86 (51.8)	
Bentall’s	46 (29.7)	48 (28.9)	
Modified Carbrol’s	30 (19.4)	32 (19.3)	
Intraoperative conditions (min)			
Procedure duration	401 ± 30	401 ± 23	0.964
CPB	187 ± 23	185 ± 21	0.317
ACC	84 ± 14	83 ± 16	0.794
HCA	20 ± 2	20 ± 2	0.385
The diameter of elephant trunk stent (mm)	28.5 ± 1.1	28.5 ± 1.0	0.774
Intraoperative hypothermia (°C)			
Minimum bladder temperature	23.3 ± 3.1	23.3 ± 3.1	0.836
Minimum nasopharyngeal temperature	21.6 ± 3.2	21.8 ± 3.1	0.632
Intraoperative blood transfusion			
RBC (U)	4.2 ± 2.1	4.5 ± 2.0	0.137
PLT (U)	0.5 ± 0.6	0.5 ± 0.6	0.780
FFP (ml)	654 ± 218	644 ± 214	0.691
Cryoprecipitation (U)	1.2 ± 1.7	1.3 ± 1.8	0.614

Note: PC: peripheral cannulation; PCC: peripheral-to-centric cannulation; CPB: cardiopulmonary bypass; ACC; aortic cross clamp; HCA: hypothermia circulatory arrest; RBC: red blood cell; PLT: platelet; FFP: fresh frozen plasma.

###  Postoperative in-hospital outcomes

**Figure 3:**
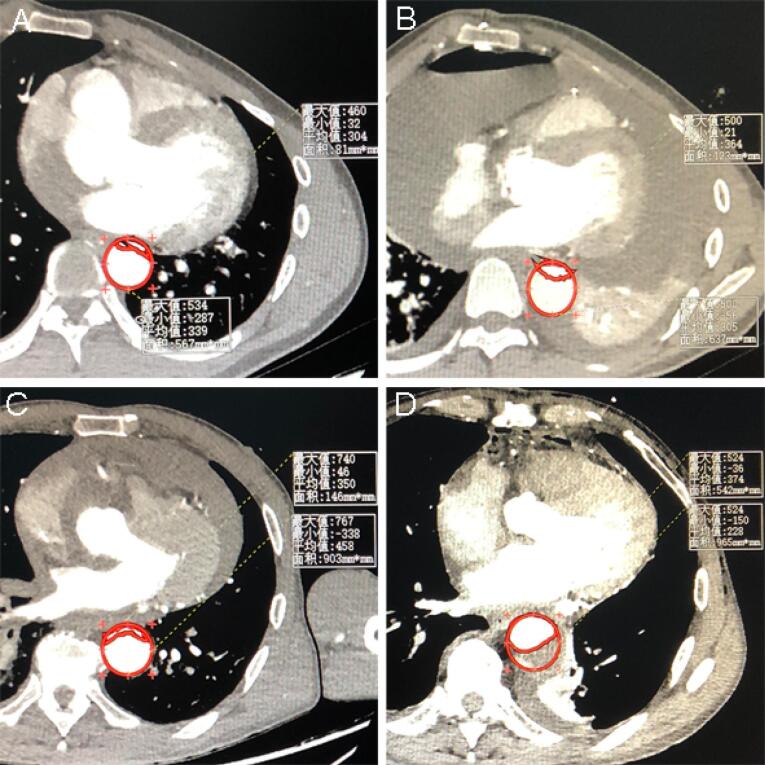
The comparison between preoperative and postoperative MFLA ratio on the descending thoracic aorta. The preoperative and postoperative MFLA ratio calculated by imaging assistant software system was 0.71 ± 0.14 (Figure 2A) and 0.44 ± 0.13 (Figure 2B) in PC group, respectively. The preoperative and postoperative MFLA ratio was 0.70 ± 0.14 (Figure 2C) and 0.36 ± 0.11 (Figure 2D) in PCC group, respectively (postoperative MFLA ratio between PC and PCC group, P < 0.001). MFLA: maximum false lumen area ratio in descending thoracic aorta; PC: peripheral cannulation; PCC: peripheral-to-centric cannulation

**Figure 4:**
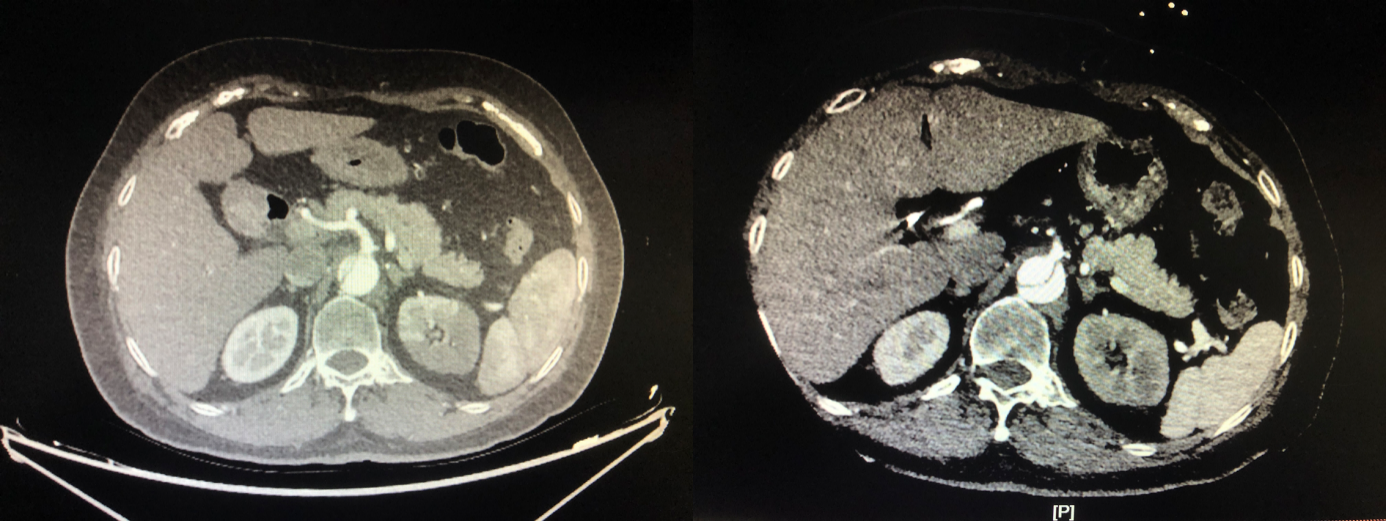
The effect on the ostium of coeliac trunk artery by PCC technique. The preoperative image indicated that the ostium was almost involved by false lumen (left column). The postoperative image indicated that the ostium was totally stemmed from true lumen (right column). PCC: peripheral-to-centric cannulation

 Multivariable logistic regression analysis showed that the perfusion method was significantly correlated with postoperative MFLA ratio. The postoperative MFLA ratio on the descending thoracic aorta were reduced more in PCC group than that in PC group with 0.35 ± 0.11 and 0.44 ± 0.13, respectively (Figure 3, *P* < 0.001). Moreover, postoperative involvement of the ostium of the dissected branch arteries on CTA including coeliac trunk artery, superior mesenteric artery, renal artery and Iliac artery was notably decreased in PCC group (Figure 4). There were a lower incidence rate of hepatic dysfunction and RRT in PCC group compared with those in PC group (15.5% vs. 39.8%, *P* < 0.001; 10.3% vs. 20.5%, *P* = 0.012, respectively). There was also a lower serum inflammation response (hs-CRP and IL-6) and anaerobic metabolism (lactate) in 24 hours after procedure in PCC group than those in PC group. The in-hospital survival, the duration of ICU and hospital stay was similar, but APACHE II score and mechanical ventilation time was lower in PCC than that in PC group, respectively (Table 3).

**Table 3:** Postoperative outcomes

	**PCC (n=155)**	**PC (n=166)**	***P*** ** value**
Systemic inflammation response			
hs-CRP (12h, mg/L)	67.5 ± 14.1	75.5 ± 12.6	< 0.001
hs-CRP (24h, mg/L)	111.8 ± 14.1	116.8 ± 15.0	0.002
hs-CRP (48h, mg/L)	186.0 ± 15.0	192.6 ± 14.7	< 0.001
IL-6 (24h, pg/ml)	104.4 ± 49.9	124.0 ± 50.1	< 0.001
Cardiac injury			
TnI (12h, ng/mL)	10.5 ± 2.5	10.8 ± 2.9	0.299
TnI (24h, ng/mL)	5.7 ± 1.3	5.6 ± 1.4	0.715
TnI (48h, ng/mL)	2.8 ± 0.9	3.0 ± 1.2	0.102
Anaerobic metabolism			
Lactate (0h, mmol/L)	6.5 ± 1.2	6.8 ± 1.2	0.013
Lactate (8h, mmol/L)	8.3 ± 1.5	8.8 ± 1.6	0.002
Lactate (16h, mmol/L)	5.0 ± 1.0	5.2 ± 1.0	0.041
Lactate (24h, mmol/L)	3.3 ± 0.8	3.4 ± 0.9	0.485
Lactate (48h, mmol/L)	2.0 ± 0.6	2.0 ± 0.7	0.922
Involved ostium of the branch arteries by intimal			
flap from CTA imaging (%)	108(69.7)		
Potent false lumen in descending thoracic aorta	0.36 ± 0.11	129(77.8)	0.127 < 0.
MFLA ratio in descending thoracic aorta	26 (16.8)	0.44 ± 0.13	001
Coeliac trunk artery	29 (18.7)	45 (27.1)	0.026
Superior mesenteric artery	75 (48.4)	47 (28.3)	0.043
Renal artery	35 (22.6)	101 (60.8)	0.025
Iliac artery	14.1 ± 3.3	57 (34.3)	0.020
CTA time after procedure		14.6 ± 3.5	0.256
ICU recovery			
APACHE II (24h)	16 ± 5	17 ± 5	0.018
Ventilation time (h)	42 ± 19	47 ± 21	0.028
Duration in ICU stay (d)	3.5 ± 1.4	3.8 ± 1.6	0.118
Chest tube drainage (ml)	949 ± 156	953 ± 160	0.825
In-hospital indicators (%)			
Death	8 (5.2)	15 (9.0)	0.179
Stroke	13 (8.4)	20 (12.0)	0.358
Paraplegia	1 (0.6)	1 (0.6)	1
Impaired liver function	24 (15.5)	66 (39.8)	*P* < 0.001
RRT (%)	16 (10.3)	34 (20.5)	0.012
Postoperative duration in hospital (d)	15 ± 4	15 ± 5	0.232
30-day mortality	10 (6.5)	16 (9.6)	0.296

Note: PC: peripheral cannulation; PCC: peripheral-to-centric cannulation; hs-CRP: high sensitivity C-reactive protein; IL-6: Interleukin-6; TnI: troponin I; CTA: computed tomography angiography; MFLA: maximum false lumen area; ICU: intensive care unit; APACHE: Acute Physiology and Chronic Health Evaluation; RRT: Renal Replacement Therapy.

## Discussion

 This study demonstrated that the resumption of centric perfusion after completing distal anastomosis was advantageous over continuous peripheral cannulation during surgical repair of AAD patients in reducing MFLA on the descending thoracic aorta from the CTA imaging. This interactive management strategy on cannulation perfusion mode was also associated with a lower inflammation response and lower incidence of hepatorenal dysfunction in AAD patients.

 Malperfusion happens up to 40% of AAD patients and contributes to increased morbidity and mortality.^11^ Choosing appropriate arterial cannulation strategy is especially pivotal on the account that distinct cannulation perfusion mode imposes a discrepant effect of the hemodynamic characteristic on the vital organ.^12^ Of several available cannulation places, the axillary artery is suggested by guidelines as the preferential cannulation choice for aortic repair in cases of AAD.^13^ The right axillary artery is commonly used for selective antegrade cerebral perfusion. However, an unsuitable cannulation could result in vertebral artery orifice obstruction or a subclavian steal phenomenon. Femoral artery cannulation or even bilaterally is usually adopted in minimally invasive cardiac surgery,^14^ which is easily available for the use in AAD patients but retrograde perfusion might increase the risk of thrombus embolization and propagation of false lumen perfusion due to flow reversal in the thoracoabdominal aorta.

 Double artery cannulation (DAC) combining right axillary artery with femoral artery on one pump is clinically applied in the repair of AAD accordingly. In this situation, femoral cannulation can enhance visceral perfusion and supply the partial blood to coeliac and superior mesenteric arteries resulting from the intimal flap motion block in the AAD model perfused only by axillary cannulation.^15^ Some known advantages include stabilization of CPB flow by the addition of axillary cannulation, prevention of the totally retrograde perfusion that occurs at single femoral artery cannulation, accelerated cooling, and perfusion of the ischemic leg if the femoral artery used for cannulation in the ischemic leg via an end-to-side anastomosed vascular prosthesis.^16^ The application of DAC don’t increase the surgical risks compared to right axillary artery cannulation, but could benefit from reducing the occurrence of postoperative acute kidney injury.^17^

 The direct aortic cannulation has been introduced in repairing AAD patients utilizing guidewire technique under transoesophageal echocardiography imaging support. Moreover, the patients using direct aortic cannulation for AAD surgery have markedly lower in-hospital mortality, paraparesis/paraplegia, mesenteric ischemia than those who had aortic arch cannulation.^18^ However, it is dangerous to be manipulated in the patients with delicate aorta wall and also risky to guarantee the certainty of positioning of the cannula into the true lumen. Similar to ascending aorta cannulation, initiation of antegrade perfusion via the proximal branch of the tetrafurcated graft avoided the potential risks of direct ascending aorta cannulation at the onset of procedure, and made use of the additional branch of artificial graft to reestablish the antegrade blood flow after the distal anastomosis was established. The one-pump double-tube technique with bifurcated perfusion lines facilitates the shift from femoral artery to centric perfusion, which could prevent retrograde perfusion flows resulting in giant false lumen.^19^ The resumption of physiological forward blood stream could reduce the limb ischemia injury on the side of femoral artery cannulation.

 High MFLA ratio is an independent index in predicting long-term proximal aortic expansion, aorta-related reintervention and even reentry tears.^20^ Preventing large residual false lumen has evolved a focus in the cardiac surgical community on treating DeBakey type I dissection. The retraction of residual false lumen could protect downstream perfusion such as kidney and abdominal organ which was sensitively affected by metabolism damage and inflammation response incurred by ischemia insult.^21^ The intimal lumen of thoracoabdominal aorta was unlikely to expand outside under the unordered, low-pressure and non-pulsatile turbulence state when the two opposing currents blend together during the period of CPB. The promptly resumed centric perfusion with continuous bloodstream provided radial expansion under the circumferential pressure and prevent elastic recoil of the dilated true lumen.^22^ Previousstudy demonstrates that the patients undergoing RRT has lower MFLA change in the upper abdominal aorta,^10^ which was reflected by the ameliorated results on the involved ostium of coeliac trunk artery and superior mesenteric artery by false lumen in PCC group.

 Hyperlactatemia has been shown to be a sensitive indicator of impaired oxygen transport and tissue hypoperfusion.^23^ In this study, the lactate level between the two groups was regarded to mainly reflect the perfusion difference from the visceral organ and lower limb due to on the same regimen at heart and brain protection.^24^ Although there was no incidence of ischemia and necrosis of ipsilateral limb, the antegrade perfusion in PCC group could reduce or ahead of time discontinue the excessive anaerobic metabolism in PC group. Through a timely centric perfusion, the incidence of liver dysfunction and RRT were less than that with the continuous peripheral perfusion mode, which was inconsistent with physiological unidirectional blood flow in the descending aorta. Although most of the acute kidney injury after repair of AAD could be recovered before discharge of hospital,^25^ a higher rate of renal atrophy was likely to happen if hypoperfusion existed,^26^ a shorter ventilation time and lower APACHE Ⅱ score in ICU benefited the patients both physically and economically.^27^

 This study has certain limitations. Firstly, it is a single-center study and of retrospective observation design. Secondly, the influence of era spanning six-year duration cannot be avoided including the alternation on laboratory analysis package, the rationality on the usage of inotropes and vasopressors, and the effect on learning curve of surgeon. Thirdly, long-term follow-up on MFLA is lacking.

## Conclusion

 This study demonstrated the perfusion mode with transition from peripheral artery-to-centric aorta artery via the perfusion branch of artificial graft during surgical repair of AAD was associated with a lower MFLA ratio in the descending thoracic aorta and lower incidence of postoperative hepatorenal impairment.

## Competing Interests

 The authors declare that they have no competing interests

## Ethical Approval

 The study was approved by Institutional Medical Ethics Committee (Approval No. 2021215). Consent to participate was waived due to retrospective nature of study.

## References

[1] Jiang Q, Du J, Yu T, Huang X, Zuo M, Huang K (2022). Ascending aortic aneurysm and dissection secondary to bicuspid aortic valve with concomitant coarctation of descending aorta successfully repaired with extracorporeal membrane oxygenation support: a case report. Cardiol Discov.

[2] Zhu Y, Lingala B, Baiocchi M, Tao JJ, Toro Arana V, Khoo JW (2020). Type A aortic dissection-experience over 5 decades: JACC historical breakthroughs in perspective. J Am Coll Cardiol.

[3] Wang Z, Ge M, Chen C, Lu L, Zhang L, Wang D (2021). Hepatic dysfunction in patients who received acute DeBakey type I aortic dissection repair surgery: incidence, risk factors, and long-term outcomes. J Cardiothorac Surg.

[4] Haddadi H, Asham O, Soleimani A (2025). Evaluation of risk factors in patients with calcific aortic valve disease who underwent aortic valve replacement from 2011 to 2021. Biomed Adv.

[5] Kreibich M, Chen Z, Rylski B, Bavaria JE, Brown CR, Branchetti E, et al. Outcome after aortic, axillary, or femoral cannulation for acute type A aortic dissection. J Thorac Cardiovasc Surg 2019;158(1):27-34.e9. doi: 10.1016/j.jtcvs.2018.11.100. 31248512

[6] Jiang Q, Liu SZ, Jiang L, Huang KL, Guo J, Hu SS (2019). Comparison of two radiofrequency ablation devices for atrial fibrillation concomitant with a rheumatic valve procedure. Chin Med J (Engl).

[7] Jiang Q, Yu T, Huang K, Liu L, Zhang X, Hu S (2018). Feasibility, safety, and short-term outcome of totally thoracoscopic mitral valve procedure. J Cardiothorac Surg.

[8] Okita Y (2021). Frozen elephant trunk usage in acute aortic dissection. Asian Cardiovasc Thorac Ann.

[9] Jiang Q, Yu T, Huang KL, Liu K, Li X, Hu SS (2024). Carotid versus axillary artery cannulation for descending aorta remodeling in type A acute aortic dissection. World J Cardiol.

[10] Jiang Q, Du J, Lei Y, Gu C, Hong L, Hu S (2023). The relationship between false-lumen area ratio and renal replacement therapy after acute aortic dissection repair on bilateral artery cannulation: a cross-sectional study. Quant Imaging Med Surg.

[11] Norton EL, Khaja MS, Williams DM, Yang B (2019). Type A aortic dissection complicated by malperfusion syndrome. Curr Opin Cardiol.

[12] Jiang Q, Huang K, Wang D, Xia J, Yu T, Hu S (2024). A comparison of bilateral and unilateral cerebral perfusion for total arch replacement surgery for non-marfan, type A aortic dissection. Perfusion.

[13] Erbel R, Aboyans V, Boileau C, Bossone E, Bartolomeo RD, Eggebrecht H (2014). 2014 ESC guidelines on the diagnosis and treatment of aortic diseases: document covering acute and chronic aortic diseases of the thoracic and abdominal aorta of the adult The Task Force for the Diagnosis and Treatment of Aortic Diseases of the European Society of Cardiology (ESC). Eur Heart J.

[14] Jiang Q, Wang Z, Guo J, Yu T, Zhang X, Hu S (2021). Retrospective comparison of endoscopic versus open procedure for mitral valve disease. J Invest Surg.

[15] Heo W, Lee GH, Kim TH, Lee Y, Huh H, Ha H (2022). Quantification of visceral perfusion and impact of femoral cannulation: in vitro model of aortic dissection. Eur J Cardiothorac Surg.

[16] Kusadokoro S, Kimura N (2022). Double arterial cannulation: a classical yet useful cannulation strategy-comment on cannulation strategy in frozen elephant trunk for type A aortic dissection: double arterial cannulation approach. Eur J Cardiothorac Surg.

[17] Zhang H, Xie W, Lu Y, Pan T, Zhou Q, Xue Y (2021). Double arterial cannulation versus right axillary artery cannulation for acute type A aortic dissection: a retrospective study. J Cardiothorac Surg.

[18] Juvonen T, Jormalainen M, Mustonen C, Demal T, Fiore A, Perrotti A (2023). Direct aortic versus supra-aortic arterial cannulation during surgery for acute type A aortic dissection. World J Surg.

[19] Liang S, Liu Y, Zhang B, Dun Y, Guo H, Qian X, et al. Cannulation strategy in frozen elephant trunk for type A aortic dissection: double arterial cannulation approach. Eur J Cardiothorac Surg 2022;62(3). doi: 10.1093/ejcts/ezac165. 35293587

[20] Kim JH, Lee SH, Lee S, Youn YN, Yoo KJ, Joo HC (2022). Role of false lumen area ratio in late aortic events after acute type I aortic dissection repair. Ann Thorac Surg.

[21] Jiang Q, Xiang B, Wang H, Huang K, Kong H, Hu S (2019). Remote ischaemic preconditioning ameliorates sinus rhythm restoration rate through Cox maze radiofrequency procedure associated with inflammation reaction reduction. Basic Res Cardiol.

[22] Osswald A, Schucht R, Schlosser T, Jánosi RA, Thielmann M, Weymann A (2021). Changes of stent-graft orientation after frozen elephant trunk treatment in aortic dissection. Eur J Cardiothorac Surg.

[23] Jiang Q, Li H, Huang X, Yu L, Lueck S, Hu S (2020). Postnatal exposure to hypobaric hypoxia and its impact on inflammation and injury indexes after a cardiac valve procedure. Interact Cardiovasc Thorac Surg.

[24] Jiang Q, Yang Y, Sun H, Tang Y, Lv F, Hu S (2020). Stable hemodynamics within “no-touch” saphenous vein graft. Ann Thorac Cardiovasc Surg.

[25] Nishio H, Sakakibara Y, Ikuno T, Seki Y, Nishimura K (2023). Impact of recovery from acute kidney injury after aortic arch repair. Ann Thorac Surg.

[26] Wang CC, Lin HS, Huang YL, Wu FZ, Chuo CC, Ju YJ (2018). Renal artery involvement in acute aortic dissection: Prevalence and impact on renal atrophy in non-interventional treatment patients. J Cardiovasc Comput Tomogr.

[27] Jiang Q, Yu T, Huang K, Huang X, Zhang Q, Hu S (2022). The impact of medical insurance reimbursement on postoperative inflammation reaction in distinct cardiac surgery from a single center. BMC Health Serv Res.

